# Correction: Park, J.; et al. Fine-Grained Power Gating Using an MRAM-CMOS Non-Volatile Flip-Flop. *Micromachines* 2019, *10*, 411

**DOI:** 10.3390/mi11010011

**Published:** 2019-12-19

**Authors:** Jaeyoung Park, Young Uk Yim

**Affiliations:** School of Computer Science and Electrical Engineering, Handong Global University, Pohang-si 37554, Korea; zero12@gmail.com

As this research is a personal achievement with no company-related information, and there is no financial support for this paper from Qualcomm Technologies Inc., the authors would like to change the affiliation, authors email addresses, and funding information in the published paper [1]. The affiliation was changed from “Qualcomm CDMA Technologies, Qualcomm Technologies Inc., San Diego, CA 92121, USA” to “School of Computer Science and Electrical Engineering, Handong Global University, Pohang-si 37554, Korea”. The email of Jaeyoung Park changed from jaeyngp@qti.qualcomm.com to jaeyoung.park@handong.edu, email of Young Uk Yim changed to zero12@gmail.com. The funding part changed from “This research received no external funding” to “This work was supported by the National Program for Excellence in Software at Handong Global University (2017-0-00130) funded by the Ministry of Science and ICT“.

The changes do not affect the scientific results. We apologize for any inconvenience caused to the readers. The manuscript will be updated, and the original version will remain available online on the article webpage, with a reference to this correction. 
